# Perinatal Outcomes and Management of Umbilical Vein Varix: A Comprehensive Review of 392 Cases

**DOI:** 10.3390/jcm14020441

**Published:** 2025-01-11

**Authors:** Taylor Ghahremani, Anna Bailey Britt, Emily Ray, Ashton Jones, Ruofei Du, Everett F. Magann

**Affiliations:** 1 Department of Obstetrics and Gynecology, University of Arkansas for Medical Sciences, 4301 W. Markham St. Slot # 518, Little Rock, AR 72205, USAkajones@uams.edu (A.J.); 2 Department of Biostatistics, University of Arkansas for Medical Sciences, Little Rock, AR 72205, USA

**Keywords:** umbilical vein varix, intrauterine fetal demise, thrombosis, fetal growth restriction

## Abstract

Case reports and case series have linked umbilical vein varices (UVVs) with adverse pregnancy outcomes. Newer case reports and series suggest better perinatal outcomes in cases with an isolated UVV. The purpose of this literature review is to determine if there is commonality in management, outcomes, and association in pregnancy with UVV and fetal aneuploidy, growth restriction, demise, thrombosis, and turbulent flow. Secondly, we will review the diagnosis, pathophysiology, differential diagnosis, and incidence of UVV. A literature search was undertaken using the search engines PubMed, CINAHL, and Embase. The search terms used were “umbilical vein” AND “varix” OR “umbilical varix” AND “fetal death” OR “fetal demise” AND “guideline” AND “outcome” OR “abnormality” OR “abnormalities”. There were 169 abstracts identified. We identified 392 cases of UVV from our literature review. There is a higher risk of fetal anomalies, fetal aneuploidy, and intrauterine fetal demise in pregnancies with UVV. The risk for fetal growth restriction is in the higher range of normal. Turbulence in and thrombosis of the UVV are concerning developments that influence fetal surveillance. Pregnancies with a UVV are more likely to have fetal anomalies, aneuploidy, and IUFD. We propose an antenatal assessment and management plan and recommendations for the timing of delivery. Our aim is to analyze existing literature to identify and combine case reports and case series of umbilical vein varix and analyze fetal and perinatal outcomes.

## 1. Introduction

An umbilical vein varix (UVV) is defined as a focal enlargement of the umbilical vein. This enlargement is defined as greater than 9 mm, more than 50% of the width compared to the non-dilated portion of the vein, or greater than two standard deviations above the mean for gestational age [1]. The varices have been identified both in fetal extra-abdominal and intra-abdominal locations.

Case reports and case series from the 1990s and early 2000s have linked the presence of intra-abdominal varices with an increased risk of chromosomal abnormalities, anatomic anomalies, intrauterine fetal demises (IUFD), fetal hydrops, and preterm deliveries [1,2,3]. Newer case reports and case series suggest better perinatal outcomes in cases with an isolated UVV [4]. The purpose of this review and evaluation of the existing literature is to determine if there is commonality in management, outcomes, and associations of UVV and fetal aneuploidy, anomalies, growth restriction, demise, turbulent flow, and thrombosis and to determine if certain characteristics of UVV warrant further antenatal surveillance and an earlier delivery. Secondly, we will review the diagnosis pathophysiology, differential diagnosis, and incidence of UVV.

## 2. Methods

**Figure 1:**
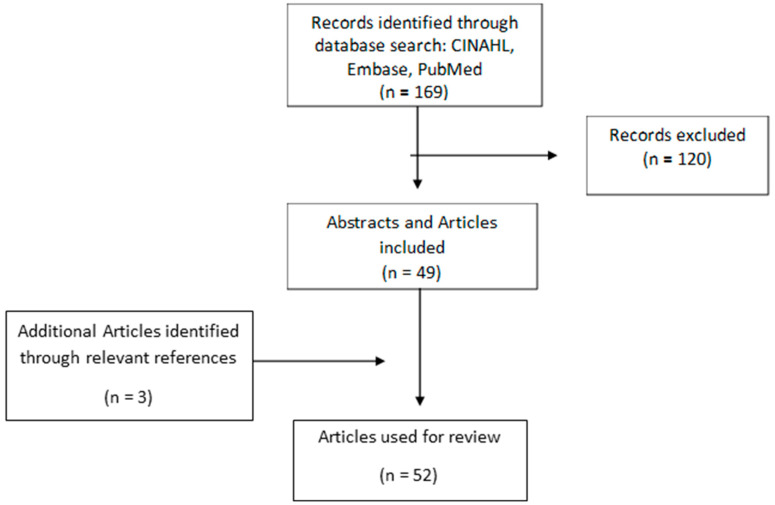
Flowchart describing the systematic review procedure.

A literature search was undertaken by our university librarian after a discussion with her about the comprehensive review we wanted to undertake on the association between the presence of an intra-abdominal UVV and intrapartum and perinatal pregnancy outcomes. The librarian used the search engines PubMed, CINAHL, and Embase. The search terms that the librarian used were “umbilical vein” AND “varix” OR “umbilical varix” AND “fetal death” OR “fetal demise” AND “guideline” AND “outcome” OR “abnormality” OR “abnormalities”. The search was limited to the English language. There were 169 abstracts identified. Three authors (TG, EFM, AB) independently reviewed all the abstracts and the full articles of these abstracts that discussed UVVs and intrapartum and perinatal outcomes. The references of these articles were reviewed for any additional relevant articles. There was no limitation put on the years searched. After reviewing the articles, 53 relevant studies were used and are the basis for this review (Figure 1).

This study reviewed intra-abdominal UVVs and intrapartum and perinatal outcomes. The outcomes of interest for this study were turbulent flow within the varix, fetal anomalies, fetal aneuploidy, fetal growth restriction (FGR), and intrauterine fetal demise (IUFD). Seven of the case series reported their results as the number of cases that had the pregnancy outcome of interest, out of the total number of cases [4,5,6,7,8,9,10]. This group had the largest number of cases collectively (*n* = 287) and is labeled “combined cases”. The other case reports were individual case reports or a series of case reports, each of which reported the individual outcomes of each case, totaling 105 cases. This group was labeled “individual cases” [1,2,3,11,12,13,14,15,16,17,18,19,20,21,22,23]. The analysis of this subgroup is informative by evaluating the associations of each individual outcome rather than the number affected out of the total group in the “combined cases”. The outcomes of this study will be presented as total cases (combined and individual cases), combined cases (large groups with outcomes reported for the whole group, and individual cases (in which the outcomes of interest are presented for each patient in the group. Additionally, we reviewed case reports with observed UVV thrombosis, associated neonatal coagulopathy, and iatrogenic UVV, all of which were rare.

## 3. Diagnosis

**Figure 2:**
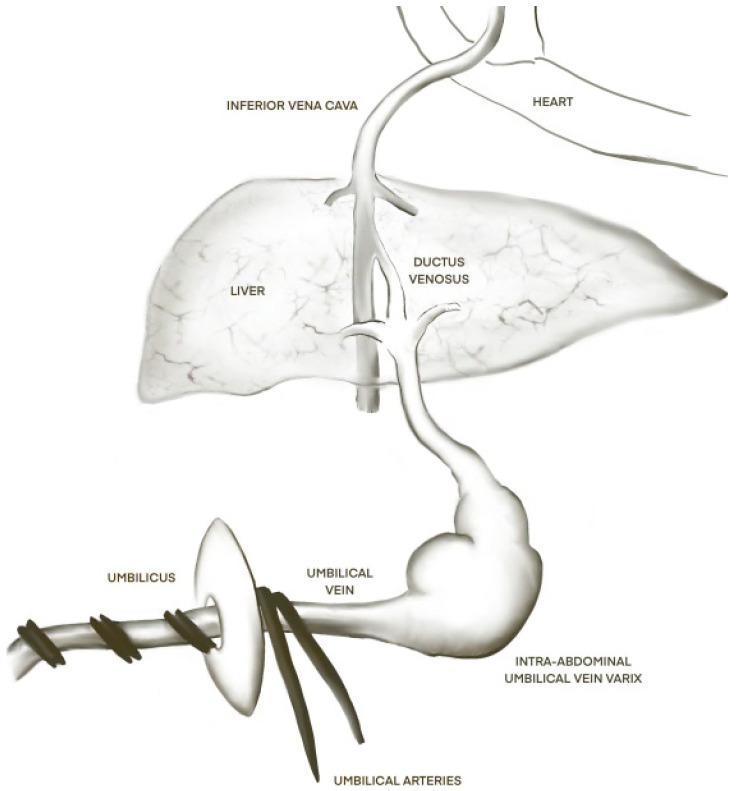
Schematic diagram of intra-abdominal UVV.

UVV is seen on ultrasound as an anechoic, oval-shaped, or rounded mass located between the abdominal wall and the lower edge of the liver as a continuation of the umbilical vascular plane [24] (Figure 2). Diagnostic criteria have classically been defined as a diameter exceeding 9 mm or a diameter of the sub-hepatic segment of the upper umbilical vein exceeding 50% of the diameter of the intra-hepatic segment [24]. One author describes the use of 3D inversion/4D color and power Doppler to map the entirety of the varix’s shape and course, stating that standard 2D sonography is inadequate [25]. In most cases, the dilation does not change on serial ultrasound examinations but may fluctuate 1 to 3 mm as the fetus grows [24]. Melcer et al. studied median hCG, AFP, and uE3 levels of women with UVV and did not observe any difference compared to controls; thus, serum screening is likely to be unhelpful for identifying those at risk for UVV early in pregnancy [26]. To confirm that the diagnosis of UVV is indeed isolated, some authors call for a detailed anatomic survey, fetal echocardiogram, and karyotyping (especially if other anomalies are found) [3,4,12]. By contrast, Bas Lando et al. note that fetal echo and karyotyping, unless otherwise indicated, is not helpful in the workup of isolated UVV and that it could potentially represent a normal variant in the absence of other findings [5].

## 4. Pathophysiology, Differential Diagnosis, and Incidence

Umbilical vein varix typically occurs in the portion of the umbilical vein between the fetal abdominal wall and the fetal liver. This intra-abdominal portion of the umbilical vein is unsupported and may make the vein susceptible to pressure changes in the vein resulting in a segmental dilation [8]. The differential diagnosis for a dilated cystic structure in this area would be a urachal cyst, a meconium pseudocyst, an ovarian cyst, a choledochal cyst, cystic lymphangioma, or an enteric duplication cyst [8]. The diagnosis is made by the demonstration of blood flow using color Doppler through the cystic area. The incidence of umbilical vein varices is estimated from case series to be 0.1–2.8/1000 deliveries [8,24]. Similar to an extra-abdominal umbilical cord cyst, the main clinical concern comes from cord compression or thrombosis leading to IUFD; however, it is difficult to predict such a catastrophic event. This is a major challenge for the clinician during the workup, counseling, and surveillance of the diagnosis.

## 5. Case Series and Case Reports

There were 23 case reports and case series of interest totaling 392 fetuses with a pregnancy complicated by a UVV and from which the intrapartum and perinatal outcomes were evaluated. For the “combined” and “individual” case reports and case series, the mean maternal age was 30.95 years (SD = 5.21). The average gestational age at diagnosis of the UVV was 29.94 weeks (SD = 4.76). The mean largest diameter of the UVV was 12.85 mm (SD = 2.79) and the average gestational age at delivery was 37.63 weeks (SD = 2.90). (Table 1) Intrapartum and perinatal outcomes were assessed from these 392 cases.

## 6. Intrapartum and Perinatal Outcomes

### 6.1. Intrauterine Growth Restriction and UVV

Of the 392 cases, 280 analyzed intrauterine growth restriction (IUGR). Eighteen of these pregnancies (18/280, 6.4%) were complicated by fetal growth restriction [2,4,5,7,8,9,15,16,21,22]. One of the UVV pregnancies with IUGR (no fetal anomalies or fetal aneuploidy) was associated with IUFD [21].

**Figure 3:**
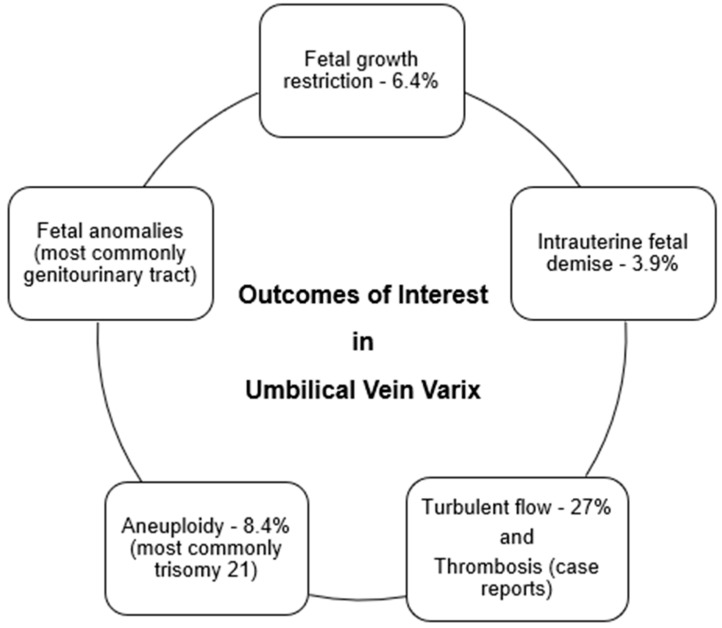
Summary of outcomes of interest in UVV.

Based on these findings, the rate of IUGR in fetuses with UVV compared to the rates of IUGR in pregnancy overall is on the higher end of the reported range of 3–7%, as noted in Figure 3 [27].

### 6.2. Fetal Anomalies and UVV

In the total cases reviewed, there were 375 cases in which fetal anomalies were mentioned as present or absent. Fetal anomalies were identified in 56 of these cases (15%) [2,3,4,5,6,7,8,10,14,15,16,21]. The most common anomalies observed were of the genitourinary tract [6].

Among single case reports, there was a case of a fetus with an absent ductus venosus, whereby the UVV flow drained directly into the IVC and ultimately caused high output cardiac failure [28]. The fetus also had other cardiac findings including a large subaortic VSD [28]. The infant died on day 1 of life secondary to cardiorespiratory failure [28]. Another case report described associated anomalies including dilated colon/rectum, urinary tract dilation, and single umbilical artery. Postnatally, the infant was diagnosed with an imperforate anus and a recto-urethral fistula [29]. Finally, one case report of UVV without any detected structural anomalies or growth disorders was diagnosed postnatally with distal epispadias [30].

According to this literature review, fetal anomalies are more common in fetuses with UVV compared to the rates of fetal anomalies in pregnancy overall (1.6% for singletons), as noted in Figure 3 [31].

### 6.3. Aneuploidy and UVV

In our combined cases review, there were 166 cases that were evaluated for fetal aneuploidy [1,2,3,4,6,10,15,16]. There were 14 cases with fetal aneuploidy (8.4%), most commonly trisomy 21 as noted in Figure 3 [1,6,15,16]. Four of the cases were terminated [3,4,13], and four cases were linked with IUFD [1,2,6].

In a case report review, a singular case report commented on a karyotype confirmation of trisomy 21 in a pregnancy with UVV and ASD [32]. Those authors advocate that detailed anatomy, fetal echocardiogram, and karyotyping are necessary to be able to rule a UVV as an isolated finding [32].

### 6.4. Turbulent Flow in UVV

In the combined cases, there were 94 cases in which the blood flow in the UVV was recorded as turbulent or not [5,7,9,12,13,16,20,23]. In 25 of these cases, the blood flow was documented as turbulent (27%) as noted in Figure 3 [5,7,9,12,16,20,23]. In the Bas–Lando series, seven of the 23 cases had turbulent flow and there were no IUFDs. In the Brenner study, seven of 14 cases had turbulent flow and there were no IUFDs. In the Di Pasquo study, one of 13 cases had turbulent flow but it could not be determined if the IUFD was related to turbulent flow or not. In the Lallar study, two of the three cases had turbulent flow, with one pregnancy being terminated because of fetal aneuploidy, and the other case did not have an IUFD. In the Valsky study, two of the seven cases had turbulent flow and there was one IUFD, but it could not be determined if the UVV had turbulent flow. In the Zael study, there was one pregnancy with turbulent flow without fetal death.

In the individual case reports, a case report of a UVV with no ultrasound-diagnosed anomalies or FGR but with a postnatal diagnosis of distal epispadias developed turbulent flow at 36 weeks, which prompted delivery [30]. Alternatively, a separate case report of a UVV diagnosed at 35 weeks in a diabetic mother showed turbulent flow, which was managed with inpatient antenatal surveillance until 37 weeks. At 37 weeks, because of the sub optimally controlled type 2 diabetes and the presence of a UVV, the patient was delivered. Postnatally, an US showed the UVV decreasing in size, but a thrombus was detected in the UVV [33].

The diagnosis of turbulent blood flow in the UVV has not been well described or defined; it is a subjective finding and thought to be a precursor of a UVV thrombosis. In our eligible combined cases, over one-fourth had turbulent flow documented with rare IUFDs. However, turbulent flow may prompt earlier delivery due to concern for impending thrombosis or IUFD, thus making it difficult to make recommendations surrounding this diagnosis.

### 6.5. Thrombosis in UVV

The diagnosis of a UVV thrombosis may be difficult on prenatal imaging. Moreover, attempting to predict if thrombosis poses a significant management problem may be also challenging. Viora et al. described a case of a UVV thrombosis at 32 weeks that prompted delivery [34]. A case report of a diamniotic, dichorionic twin pregnancy from Japan described inpatient antenatal surveillance starting at 34 weeks following an IUFD noted in one twin at 35 weeks + 6 days without a known cause [35]. Following the demise, the ultrasound showed an extra-abdominal UVV with thrombus near the placental cord insertion site of the surviving co-twin, who was delivered urgently and survived [35]. Another case report described a UVV that was imaged postnatally with ultrasound and found to have thrombosis that extended into the portal vein [36]. This prompted a thrombophilia workup, which revealed a factor V heterozygous mutation in the neonate’s father, and the neonate was also affected [36]. Similarly, one case report commented on a UVV and velamentous cord insertion diagnosed at 28 weeks [37]. At 31 weeks, the UVV had increased in size and there was evidence of thrombosis. Delivery occurred at 32 weeks, and a neonatal abdominal ultrasound revealed an extensive thrombosis from the umbilical vein to the portal vein. The infant died despite anticoagulation therapy. The fetal autopsy was not available for review [37]. Allen et al. described a case of a probable congenital varicella infection with a concomitant UVV with thrombosis diagnosed at 31 weeks [38]. The pregnancy was managed until 36 weeks. Postnatal imaging revealed thrombosis from the umbilical vein and into a dilated left portal vein [38].

Thrombosis of the umbilical vein is not well studied; hence, there are no specific guideline-driven tactics for diagnosis and management and no consensus on immediate management of UVV thrombosis [39]. As described, in some cases the provider opted for delivery upon first diagnosis, and others have managed the pregnancy beyond this finding. Because thrombosis is thought to be a cause of IUFD in UVV cases, the shared decision-making and risk–benefit analysis must be weighed against the risks of prematurity.

The postnatal findings of thrombosis may or may not coincide with prenatal ultrasound findings. Therefore, it is reasonable to suggest postnatal imaging of the umbilical/portal venous system after the antenatal diagnosis of a UVV.

## 7. Intrauterine Fetal Demise and UVV

In our analysis of the 392 cases (combined and individual cases) there were three cases with fetal aneuploidy that were terminated and one case where the IUFD was thought to be the result of isoimmunization leaving 388 cases (285 combined and 103 individual) for analysis [3,4]. There were 15 cases with an IUFD (3.86%) in the 388 cases. In the “combined cases” (285) there were four IUFDs (1.04%). In the combined cases one of the four IUFDs was associated with trisomy 21. No other associations were possible because of the combining of the analysis.

In the “individual” 103 cases there were 11 IUFDs (10.68%). In the two IUFDs reported by Fung, one was associated with fetal aneuploidy and fetal anomalies, and the other was not associated with turbulent flow, anomalies, aneuploidy, or FGR. One of the four cases of IUFD by Mahoney, was associated with fetal aneuploidy and no associations were found in the other three. In the two IUFDs of Sepulveda, one was associated with aneuploidy and IUGR and the other with anomalies, aneuploidy, and IUGR. In the two IUFDs reported by Valsky, turbulent flow only was observed in one of the IUFDs and the other IUFD had no associations. In the one IUFD reported by Viora there was FGR. In summation, of the 11 cases with an IUFD, six had an association with turbulent flow, anomalies, aneuploidy or IUGR.

Therefore, the overall risk of IUFD is higher among fetuses with UVV compared to the overall rate of IUFD in pregnancy (0.57%) [40]. However, almost half of the IUFDs with UVV were associated with another concerning finding including FGR, aneuploidy, turbulent flow, or other fetal anomalies. It is reasonable to conclude that having a concurrent abnormal finding may be associated with IUFD in UVV pregnancies. Conversely, it appears an increased risk of IUFD overall exists, even if these other findings are not present. A more recent retrospective cohort study evaluated singleton fetuses between 2007 and 2023 with isolated UVVs from the fetal medicine units of Amsterdam UMC. The cohort included 43 singleton pregnancies. The investigators observed no increased risk of IUFD up to 39 weeks, but a possible association of fetal growth restrictions compared to the pooled literature [41].

## 8. Other UVV Considerations

In addition to UVV thrombosis, neonatal coagulopathy has also been observed in association with UVV. One case report describes a UVV in a premature infant who was small for gestational age (SGA) [42]. The infant developed severe consumptive coagulopathy, which resolved as the varix decreased in size over time [42]. The infant developed cerebellar hemorrhage noted on MRI. Another study reported a case of selective IUGR in a monochorionic diamniotic twin pregnancy at 27 weeks. At 29 weeks, twin to twin transfusion was diagnosed, with the recipient twin having a UVV. Both fetuses had normal karyotypes via amniocentesis. Ultimately the pregnancy was delivered at 31 weeks for a non-reassuring fetal heart rate. The twin with the UVV had a coagulopathy in the neonatal period. Dual survivorship was documented [43]. Another case report of UVV diagnosed at 35 weeks had turbulent flow within the varix and thrombosis. The infant developed consumptive coagulopathy but ultimately did well [44]. In a literature survey, coagulopathy was reported in four out of 15 infants with UVVs born at less than 34 weeks [42]. This has been postulated to be related to the prematurity of the coagulation cascade or perhaps is a sequela of progressive thrombosis [42]. Therefore, coagulation studies should be considered in preterm infants with UVV, especially if thrombosis is present.

Another consideration would be the development of an iatrogenic UVV. A case report describes a patient who presented at 26 weeks with fetal hydrops and growth restriction, with elevated MCA Dopplers to 2.2 MoM [45]. Cordocentesis confirmed anemia. Hydrops resolved 7 days after transfusion, however repeat US showed new extra abdominal UVV with marked turbulent flow through a 13 mm diameter dilatation [45]. Fetus delivered at 33 weeks’ gestation via CS due to non-reassuring fetal status. Pathological analysis revealed a focal vascular smooth muscle tear of the umbilical vein [45]. It is probable that the cordocentesis caused a smooth muscle injury to the umbilical vein, causing an aneurysmal-like dilatation and resultant UVV. This singular case report brings awareness to the potential for iatrogenic UVV.

## 9. Management Strategies Used in the Case Reports and Case Series

Optimal antenatal management of isolated UVV is unknown and no strict guidelines exist. Bas Lando et al. suggest merely following the growth of the fetus [5]. Lee et al. performed an anatomic survey and high-resolution ultrasound with Doppler, and weekly nonstress test [8]. If the diagnosis is made before 32 weeks, a follow up ultrasound should occur between 32–36 weeks [8]. Induction of labor or cesarean delivery was not performed strictly on the basis of UVV diagnosis or size [8]. Brenner et al. suggest weekly Doppler of the UVV from diagnosis until 28 weeks, then twice weekly after that, with delivery at 36–37 weeks unless indicated sooner by fetal compromise [12]. Novoa et al. recommends that if the UVV appears isolated, fetal growth and UVV size should be examined every 4 weeks, with consideration of BPP surveillance more frequently after 32 weeks [4]. Di Pasquo et al. suggested that an increase in antenatal surveillance be initiated after 32 weeks, although it is not clear whether this could have helped avoid an IUFD [7]. Cohort studies are needed to compare antenatal surveillance versus expectant management [7]. Until then, di Pasquo et al. recommends ultrasound every 2 weeks starting at 32 weeks to look for signs of fetal compromise or UVV thrombus, with delivery occurring at that time after the consideration of gestational age and risks of prematurity [7]. Byers et al. initiated antenatal surveillance at 32 weeks (twice weekly NSTs, weekly AFI measurement) [6]. Delivery was indicated based on non-reassuring fetal heart rate tracing in 19% of cases, and delivery was achieved by 40 weeks [6]. One center in Italy managed four cases of fetal UVV with maternal low dose aspirin until 35 weeks with reported good outcomes, although no randomized trials exist to confirm its efficacy at prevention of thrombosis [46]. In summary, it is reasonable to perform ultrasounds to evaluate the UVV every 1 to 2 weeks and offer further fetal surveillance with non-stress test or BPP.

Does the gestational age at diagnosis play a role in outcomes? Among the combined cases, the mean gestational age at diagnosis was 29.94 weeks (SD 4.76). In Brenner’s case series, there was no difference in outcomes in those with UVV diagnosed before or after 28 weeks [12]. There were no cases of turbulent flow in those diagnosed after 28 weeks [12]. In the individual cases, there were several cases of UVV diagnosed in the third trimester [17,23,47,48,49]. One was associated with growth restriction, two with either glucose intolerance or gestational diabetes [23,47,48]. All delivered in the late preterm or early term period with good outcomes. One author notes that the peak incidence of IUFD from UVV thrombosis or rupture occurs at 27–30 weeks among available literature, citing peak fetal blood flow increase [17]. With this logic they postulate that perhaps UVV diagnosed later in pregnancy may not have a significant clinical risk, as was noted in this case series [17].

A low cerebroplacental ratio (CPR) is reported as an indicator for adverse perinatal outcomes, typically indicating placental insufficiency. In a study by Grin, a significantly higher CPR was noted in the UVV group compared to matched controls (*p* < 0.05) [50]. This reinforces the notion that adverse outcomes related to UVV are not likely to be related to a chronic issue but rather an acute event, such as thrombosis.

Given these complexities, establishing a standardized management plan is difficult. Given the increased incidence of IUFD, it is reasonable to suggest a baseline management strategy to include weekly non-stress test or biophysical profile starting by 32 weeks, and a limited ultrasound to evaluate the UVV for turbulent flow or thrombosis every 1 to 2 weeks, once a gestational age is reached at which time monitoring or delivery would be acceptable. Delivery should be offered by 39 weeks unless indicated sooner. It may be reasonable to offer early term delivery. Of course, other factors must be taken into consideration such as estimated fetal weight, presence of other anomalies, maternal conditions, etc., and a shared decision-making approach should be taken.

Although a more widely accepted surveillance strategy would be helpful, it is important to note that as previously discussed, fetal demise related to isolated UVV is thought to be the result of an acute event, such as an umbilical vein thrombosis. Antenatal testing was designed to detect uteroplacental insufficiency and fetal acidosis, thus it may not be helpful for predicting an acute, catastrophic event. Despite this, especially with fetal growth restriction, antenatal testing is warranted.

Postnatal management is not well described either. Bas Lando remarks to take postnatal considerations, such as if umbilical vein catheterization is indicated, it should be performed with caution [5]. Others have made a case for consideration of postnatal imaging of the neonate, citing that a UVV thrombus may not be detected on prenatal imaging, but rather postnatally [33]. Alternatively, one case report of what was thought to be a UVV prenatally, was an extrahepatic persistent vitelline vein, with an internal thrombus present that was surgically excised in the neonate [51].

In the combined cases, the average gestational age at delivery was 37.63 weeks (SD 2.90). Bas Lando et al. noted a 4% rate of small for gestational age infants with UVV [5]. Brenner et al. noted that turbulent flow in the UVV was associated with earlier gestational age at delivery, and smaller birthweight [12]. Fung’s case series had only one small for gestational age infant out of 13 cases [15].

Despite a lack of clear delivery guidelines, induction of labor is common in pregnancies complicated by UVV. The induction of labor rate was 65% in the Bas–Lando series, with 43% of them being the result of UVV alone [5]. Only 13.5% of IOL indications were due to UVV in the Byers case series, however all cases were delivered by 40 weeks gestation [6]. Brenner noted an increased rate of induction with turbulent flow documented within the UVV [12].

In the study by Bas–Lando et al., preterm induction for UVV posed a significantly increased rate of cesarean delivery (*p* = 0.015) and neonatal morbidity (*p* = 0.029) [5]. Rahemtullah et al. had a 13% rate of preterm delivery [3]. Novoa noted all preterm births were spontaneous [4].

Bas Lando et al. reported a cesarean delivery rate of 17% for UVV cases, however this was not related to gestational age at diagnosis [5]. Brenner had five emergent cesarean deliveries [12]. Laller et al. reported two elective cesarean deliveries at 37 weeks [16]. Novoa et al. reported on 20 cases of which one had an IUFD and two were terminated, nine were delivered vaginally (45%), seven of them being spontaneous, and seven cesarean deliveries, two were emergent, three were prescheduled, and two were the result of failed induction of labor. There was no information on the mode of delivery in the 20th patient. Brenner cited an increased risk of emergency cesarean delivery with turbulent flow seen in the UVV [12]. These studies suggest a trend associated with UVV and increased risk of cesarean delivery [4,5,12].

## 10. Long-Term Outcomes in Children with Prenatally Diagnosed UVV

We identified a single study from Israel that compared 36 children with a diagnosis of UVV with 108 controls from the same hospital without UVV and matched for gestational age at birth [10]. Characteristics of developmental delay were present in 15/36 (41.7%) of the UVV group compared to 4/108 (3.7% (*p* < 0.05)) of the control cohort [10]. However, six (16.7%) cases of UVV had other anomalies on ultrasound, and 26 (72.2%) resulted in preterm delivery, which could be a confounding factor in their developmental delay [10]. The authors of this study suggested an association between an intra-abdominal UVV and childhood developmental delay, but this association was met with the criticism that this risk may not be accurate or suitable for patient counseling [10,52]. This is likely due to the high rate of prematurity of the infants in the study and its association with developmental delays. More studies are needed to ascertain the long-term neurodevelopmental outcomes of neonates affected by prenatal UVV.

## 11. Summary

**Figure 4:**
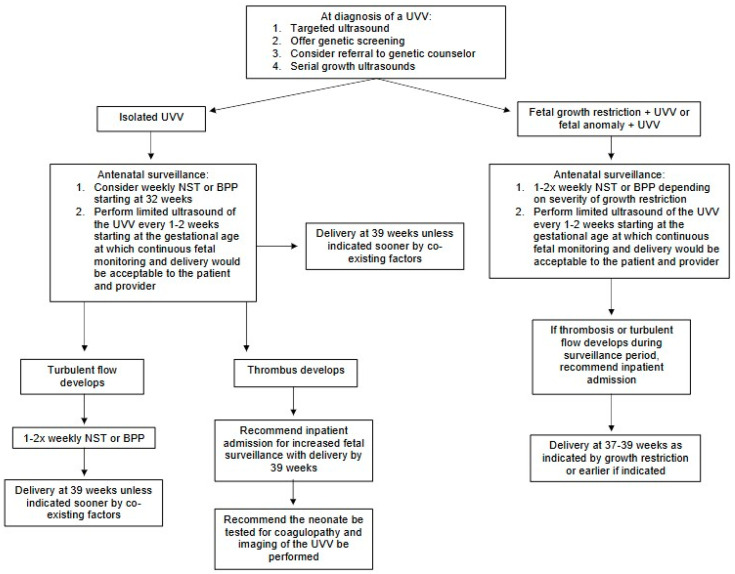
Summary of Management Considerations. Abbreviations: umbilical vein varix (UVV), non-stress test (NST), biophysical profile (BPP).

Initial management after the initial diagnosis of a UVV, if not already carried out, would be a targeted ultrasound evaluating the fetus for structural anomalies and soft markers for fetal aneuploidy (Figure 4). Additionally, if the patient has not already had genetic screening and/or genetic counseling for fetal aneuploidy, this should be offered. Serial growth ultrasounds should be performed because of the increased risk for fetal growth restriction. Pregnancies with a UVV that have one or more of the following should be considered high risk for adverse perinatal outcomes, and patients should thus be advised to pursue antenatal testing as outlined below: fetal growth restriction, aneuploidy, and other anomalies. If fetal thrombosis develops, or if there is turbulent flow present in the UVV, then additional antenatal fetal surveillance should be considered (weekly nonstress tests or biophysical profiles). If fetal growth restriction is present, antenatal testing would be performed once or twice weekly depending on the degree of growth restriction (<10% vs. <3%). Combinations of thrombosis, turbulent flow with growth restriction, or non-lethal anomalies or aneuploidy may necessitate in-hospital and daily or more frequent monitoring. Delivery should be at 39 weeks and the extent of the thrombosis, the turbulence of the flow, or the results of antenatal testing may require delivery at an earlier gestational age depending on the combination of antenatal testing, the presence of thrombosis or turbulent flow, and the presence of non-lethal anomalies and/or non-lethal aneuploidy.

**Figure 5:**
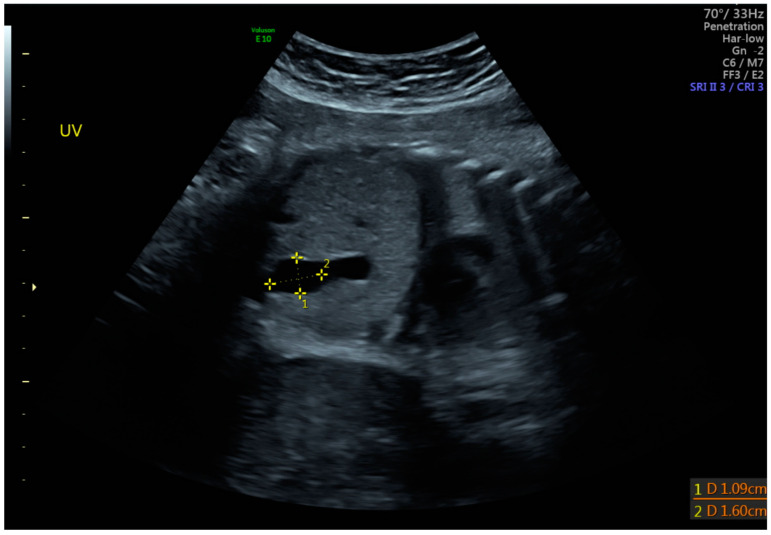
Ultrasound demonstrating measurements of an umbilical vein varix with gray scale.

**Figure 6:**
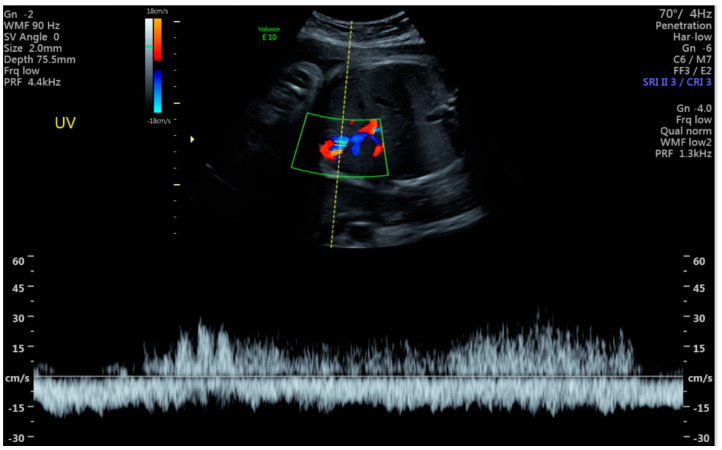
Ultrasound demonstrating an umbilical vein varix with color Doppler.

In the patient with an isolated UVV (no other anomalies, no aneuploidy, appropriate growth), based on the review of the existing literature and the increased risks for adverse events, initiation of weekly antenatal testing should be considered by the 32nd week of gestation. Limited ultrasounds to assess the UVV for turbulent flow or thrombosis should be performed weekly or every two weeks starting at a gestational age at which continuous fetal monitoring and delivery would be acceptable to the patient and provider (see Figure 5 and Figure 6).

If turbulent flow is suspected, providers should increase antenatal surveillance to twice weekly and strongly consider delivery at 39 weeks. If thrombosis is suspected, we recommend inpatient fetal surveillance with delivery by term. If thrombosis is suspected, the neonate should be tested for coagulopathy and undergo postnatal imaging of the UVV if that is possible. Lastly, an ultrasound to screen for iatrogenic extra-abdominal UVV should be performed following cordocentesis.

## Figures and Tables

**Table 1:** Demographics for Combined Cases.

Variables	Mean	Standard Deviation
Maternal age (years)	30.95	5.21
Gravidity	3.35	2.27
Gestational age at diagnosis (weeks)	29.94	4.76
Gestational age at delivery (weeks)	37.63	2.90
Largest diameter (mm)	12.85	2.79
